# Hypovirulence of *Colletotrichum gloesporioides* Associated with dsRNA Mycovirus Isolated from a Mango Orchard in Thailand

**DOI:** 10.3390/v14091921

**Published:** 2022-08-30

**Authors:** Aditya R. Suharto, Jiraporn Jirakkakul, Ana Eusebio-Cope, Lakha Salaipeth

**Affiliations:** 1 Natural Resource Management and Sustainability Program, School of Bioresources and Technology, King Mongkut’s University of Technology Thonburi, Bangkuntien, Bangkok 10150, Thailand; 2 Fungal Biotechnology Laboratory, Pilot Plant Development and Training Institute, King Mongkut’s University of Technology Thonburi, Bangkuntien, Bangkok 10150, Thailand; 3 Fit for Future Genetic Resources Cluster, Rice Breeding Innovations Platform, International Rice Research Institute, UP Los Baños, College, Los Banos 4030, Philippines; 4 LigniTech-Lignin Technology Research Group, School of Bioresources and Technology, King Mongkut’s University of Technology Thonburi, Bangkuntien, Bangkok 10150, Thailand

**Keywords:** anthracnose disease, biological control, *Colletotrichum gloeosporioides*, hypovirulence, mycovirus

## Abstract

The pathogenic fungus *Colletotrichum gloeosporioides* causes anthracnose disease, which is an important fungal disease affecting the production of numerous crops around the world. The presence of mycoviruses, however, may have an impact on the pathogenicity of the fungal host. Here, we describe a double-stranded RNA (dsRNA) mycovirus, which was isolated from a field strain of *C. gloeosporioides*, Ssa-44.1. The 2939 bp genome sequence comprises two open reading frames (ORFs) that encode for a putative protein and RNA-dependent RNA polymerase (RdRp). The Ssa-44.1 mycovirus is a member of the unclassified mycovirus family named Colletotrichum gloeosporioides RNA virus 1 strain Ssa-44.1 (CgRV1-Ssa-44.1), which has a phylogenetic similarity to Colletotrichum gleosporioides RNA virus 1 (CgRV1), which was isolated from citrus leaves in China. In *C. gloeosporioides*, CgRV1-Ssa-44.1 was shown to be linked to hypovirulence. CgRV1-Ssa-44.1 has a low spore transfer efficiency but can successfully spread horizontally to isogenic virus-free isolates. Furthermore, CgRV1-Ssa-44.1 had a strong biological control impact on *C. gloeosporioides* on mango plants. This study is the first to describe a hypovirulence-associated mycovirus infecting *C. gloeosporioides*, which has the potential to assist with anthracnose disease biological management.

## 1. Introduction

*Colletotrichum gloeosporioides*, the causal agent of anthracnose disease, is one of the most devastating diseases around the world, infecting more than 400 varieties of fruits and vegetable crops [1,2]. *C. gloeosporioides* conidia disseminate by wind and rain and are able to penetrate to the trees with the help of appressorium [3]. In Thailand, mangoes (*Mangifera indica* Linn) especially the Nam Dok Mai cultivar, are important fruits in both domestic and export markets [4]. However, mango production was affected by the infection of anthracnose disease which destroyed mangoes [5]. *C. gloeosporioides* has a broad species complex with 22 species and one subspecies [6]. On culture media, the fungus typically grows circular, wooly, or cottony colonies with a distinct color, such as pale brown or grayish white depending on their hosts [7]. *C. gloeosporioides* is known to have a hemibiotrophic lifestyle during infection on a host plant [8]. The infection of *C. gloeosporioides* is controlled by various virulence factors including *pelB* (pectate lyase B) [9], *pnl-1* (pectin lyase) [10], and *cgNPG1* (a novel pathogenic gene 1) [11]. These virulence factors contribute to the production of the plant cell wall degradation enzyme, mycelial growth, conidiation, and invasive structure development during fungal and plant host interaction [9,10,11]. In recent years, studies have searched for the most effective method to control this disease. Various methods such as physical barriers [12], the use of chemicals [3], and a combination of chemical and biological control [13] have been used. Currently, fungicide spray is the most effective control method but it poses environmental and health concerns [13]. Therefore, an alternative control technique that is environmentally friendly, such as biological control was studied and developed to solve this problem [14,15]. In this regard, the use of mycoviruses as biological control agents could be a viable option due to their successful applications such as in the case of chestnut blight fungus (*Cryphonectria parasitica*), white root rot fungus (*Rosellinia necatrix*), and a notorious plant fungal pathogen (*Sclerotinia sclerotiorum*) [16,17,18,19].

Mycoviruses are widespread in all major fungal species and are usually transmitted intracellularly during cell division, sporogenesis, and cell fusion [20]. The presence of double-stranded (ds) RNA is a typical indicator of mycovirus infection. There are seven families and one genus of ds mycoviruses [21]. Mycoviruses can neither have a physical effect on the host nor generate phenotypic changes or affect the host’s growth or physiology, potentially resulting in fungal virulence attenuation (hypovirulence) or increase (hypervirulence) [22]. Mycoviruses must be capable of lowering the fitness of the pathogenic fungus that they infect, and they must be able to transport the dsRNA efficiently enough to be maintained in a high proportion of the fungal population as potential biological control agents. The transmission of mycoviruses occurs intracellularly through hyphal anastomosis and sporogenesis. Transmission through hyphal fusion occurs naturally [23]. Meanwhile, transmission through asexual spore varieties was dependent on the virus host and spore types of the fungus [24]. 

To date, several mycoviruses have been isolated and identified from *C. gloeosporioides* such as Colletotrichum gloeosporioides URM 4903, an unidentified dsRNA mycovirus strain isolated from the cashew tree, showing isometric viral particles [25]. Colletotrichum gloeosprioides chrysovirus 1 (CgCV1), a novel dsRNA mycovirus with three dsRNA genome segments belonging to the *Chrysovirida*e family, was isolated from diseased citrus fruit [26]. A novel mycovirus isolated from cotton leaf was identified as Colletotrichum gloeosporioides ourmia-like virus 1 (CgOLV1) [27]. Colletotrichum gloeosporioides RNA virus 1 (CgRV1), a novel dsRNA mycovirus, was isolated from citrus leaves [28]. The above-mentioned mycoviruses from *C. gloeosporioides* were only characterized at the molecular level and reports on their biological properties are lacking. Thus, in this study, we investigated a large number of *C. gloeosporioides* strains from an anthracnose-infected mango leaf in Thailand Saraburi province for the presence of mycoviruses and assessed the effects they might have on their host. As a result of this research, we report here a mycovirus isolated from *C. gloeosporioides* strain Ssa-44.1, which we have designated as Colletotrichum gloeosporioides RNA virus 1 strain Ssa-44.1 (CgRV1-Ssa-44.1). CgRV1-Ssa-44.1 has a similar genomic organization to Colletotrichum gloeosporioides RNA virus 1 (CgRV1), which is an unassigned dsRNA mycovirus and demonstrates the closest phylogenetic affinity to them. We demonstrate here for the first time the biological effects of mycovirus on *C. gloeosporioides*. CgRV1-Ssa-44.1 infection on *C. gloeosporioides* reduces hyphal development, pathogenicity, and sporulation level. As a result, CgRV1-Ssa-44.1 could be a potential biological control agent for the anthracnose disease caused by *C. gloeosporioides.*

## 2. Materials and Methods

### 2.1. Fungal Isolates, Growth Conditions, and Taxonomic Analysis

*Colletotrichum gloeosporioides* strain Ssa-44.1 was originally isolated from a mango leaf in Saraburi province of Thailand that was infected by anthracnose disease. Strains Ssa-44.1 was grown on potato dextrose agar (PDA; HiMedia, Thane West, India) at 25 °C for 5 days. The total genomic DNA of strain Ssa-44.1 was isolated using the DNeasy Power Soil Pro Kit (Qiagen, Germantown, MD, USA). The Internal Transcription Spacer (ITS region of the fungal isolate was amplified by polymerase chain reaction (PCR) amplification with the universal primer pair ITS1 and ITS4 (Supplementary Table S1). Amplified DNA was purified using GenepHlow Gel/PCR kit (Geneaid, Taiwan, China) and sent for sequencing. The fungal sequence was analyzed using the BLAST program at https://blast.ncbi.nlm.nih.gov/ (accessed on 1 April 2019). Sequences were aligned by ClustalW using MEGA7 version 7.0.26 software (Pennsylvania State University, State College, PA, USA). The phylogenetic tree was constructed based on the maximum likelihood method and was tested with 1000 bootstrap replications.

### 2.2. Extraction of dsRNA from Fungal Mycelium

A modified approach was used to extract dsRNA without using phenol-chloroform [29]. The liquid nitrogen-grounded mycelia were homogenized with EBA buffer (5 mM Tris-HCl, pH 8.5; 50 mM EDTA pH 8.0; 3% SDS; 1% Polyvinyl polypyrrolidone (PVPP) and 50 mM DTT). The mixtures were centrifuged at 12,000 rpm at 4 °C for 15 min. Supernatants were collected and incubated with cellulose powder (Sigma-Aldrich, Dorset, UK) in STE buffer (10 mM Tris-HCl, pH 8.0; 150 mM NaCl and 1 mM EDTA) containing 16% (*v*/*v*) ethanol for 1 h. dsRNA was eluted with STE buffer and precipitated with absolute ethanol after three washes in STE—16%ethanol. The dsRNAs were analyzed by 1% agarose gel electrophoresis in 1× TAE buffer (Tris/acetic acid/EDTA).

Ribosomal RNAs and DNAs were then removed from the dsRNA preparation. Two hundred ng of dsRNA was digested with 2 U DNase I (New England Biolabs, Ipswich, MA, USA) at 37 °C for 1 h and subsequently treated with phenol/chloroform/isoamyl alcohol (25:24:1) (phenol saturated with water, pH 5.2), precipitated with ethanol and dissolved in diethylpyrocarbonate (DEPC)-treated water. The dsRNA was treated with 10 U S1 nuclease (Thermo Fisher Scientific, Waltham, MA, USA) and then treated with phenol/chloroform/isoamyl alcohol again and dissolved in diethylpyrocarbonate (DEPC)-treated water. The isolated dsRNA was fractionated on a 1% agarose gel and stained with ethidium bromide. dsRNA was extracted and purified using a Zymoclean Gel RNA Recovery Kit (Zymo Research, Irvine, CA, USA), before being dissolved in DEPC-treated water and kept at −80 °C until use.

### 2.3. Full-Length Analysis by Next-Generation Sequencing (NGS) Approach and RACE

Purified dsRNA (4 μg) was used for the next generation sequencing library preparation according to the manufacturer’s protocol. First strand cDNA was synthesized using ProtoScript II Reverse Transcriptase and the second-strand cDNA was synthesized using Second Strand Synthesis Enzyme Mix. The ds cDNA was purified further using magnetic beads and then treated with End Prep Enzyme Mix to repair both ends and add a dA-tail in one reaction, followed by a T-A ligation to add adaptors to both ends. Size selection of the adaptor-ligated DNA was then performed using beads, and fragments of ~400 bp (with the approximate insert size of 300 bp) were recovered. Each sample was then amplified by PCR using P5 and P7 primers, with both primers carrying sequences which can anneal to flow cells to perform bridge PCR and P5/P7 primer carrying indexes enabling multiplexing. The PCR products were cleaned up using beads, validated using an Qsep100 (Bioptic, Taiwan, China), and quantified by Qubit3.0 Fluorometer (Invitrogen, Carlsbad, CA, USA). Then libraries with different indices were multiplexed and loaded on an Illumina HiSeq/Novaseq instrument (Illumina, San Diego, CA, USA) or a MGI2000 instrument according to manufacturer’s instructions (MGI, Shenzhen, China) according to manufacturer’s instructions. Sequencing was carried out using a 2 × 150 paired-end (PE) configuration; image analysis and base calling were conducted by the HiSeq Control Software (HCS) + OLB + GAPipeline-1.6 (Illumina) on the HiSeq instrument image analysis and base calling were conducted by the NovaSeq Control Software (NCS) + OLB + GAPipeline- 1.6 (Illumina) on the NovaSeq instrument and image analysis and base calling were conducted by the Zebeacall on the MGI2000 instrument. Raw sequence quality was checked using FSATP. Clean sequences were then assembled by SPAdes software to generate contigs. The contigs were subsequently subjected to the BLAST program on NCBI. 

The 5′- and 3′-termini sequences were further determined using RACE-PCR. The total RNA of strain Ssa-44.1 was purified using the Monarch total RNA miniprep kit (New England Biolabs, USA) and used as a template. RACE-PCR of 5′- and 3′-termini was carried out following the protocol provided with a 5′/3′ RACE kit, 2nd Generation (Roche, Sigma-Aldrich, Dorset, UK). The specific primers used in this study are listed in Supplementary Table S1. The amplified cDNA products were sequenced and assembled. RT-PCR amplification with specific primers of ORF 1 and ORF 2 (Supplementary Table S1) was used to confirm the full-length sequences of the dsRNA. The dsRNA was used as a template. First-strand cDNA was synthesized with ReverstAid First Strand cDNA Synthesis Kit (Thermo Fisher Scientific, USA) and then using KAPA HiFi HotStart PCR Kit (Sigma-Aldrich, Dorset, UK) for PCR amplification.

### 2.4. Bioinformatic and Phylogenetic Analysis

The nucleotide sequences obtained were subjected to the BLAST program on the NCBI website to assess sequence similarity. Potential ORFs were deduced and translated using the DNAMAN software package with the default parameters and the ORF finder on the NCBI website. Multiple sequence alignments of nucleotide and amino acid sequences were generated using the CLUSTAL_X program. The phylogenetic trees for RdRps were constructed using the Maximum Likelihood method of MEGA7 version 7.0.26 software (Pennsylvania State University, State College, PA, USA) with bootstrapping analysis of 1000 replicates.

### 2.5. Elimination of C. gloeosporioides Strain Ssa-44.1 dsRNA

Mycelial plugs of the *C. gloeosporioides* strain Ssa-44.1 were grown on PDA at 25 °C under a 24-h photoperiod for 10 days until sporulation. The conidia were collected with 0.15% Tween 20 and cultured on PDA plates at 25 °C for 2 days. The developed colonies were separately transferred to new PDA plates for dsRNA extraction. The extracted dsRNAs were analyzed by 1% agarose gel electrophoresis, visualized by staining with ethidium bromide, and then further identified by RT-PCR. RT-PCR amplification was performed using specific primer pairs derived from the ORF 1 and 2 sequence generating 509 bp and 805 bp fragments, respectively. An annealing temperature of 60 °C was used with PCR amplification (Gene Amp PCR System 9700, Applied Biosystems, Waltham, MA, USA).

### 2.6. Virus Transmission

#### Horizontal Transmission

The virus was transferred from an infected fungal strain to a virus-free C. gloeosporioides strain Ssa-44.1#18 using the hyphal anastomosis method described by Chiba et al. [30]. Strain Ssa-44.1#18 was used as the recipient and the virus-infected strain Ssa-44.1 as the donor during hyphal anastomosis. On the same PDA plate, the donor and recipient strains were co-inoculated and cultured for 5 days at 25 °C. Three fused colonies were incubated as above and used as a source of dsRNA which was extracted and electrophoresed on a 1% agarose gel in 0.5× TAE buffer (Tris/acetic acid/EDTA).2.6.2. Vertical Transmission.

Vertical transmission of *C. gloeosporioides* strain Ssa-44.1 was measured based on conidial sporulation as described by Eusebio-Cope [31]. Conidia were harvested from *C. gloeosporioides* strain Ssa-44.1 as described above and spores were allowed to germinate. At least 30 germinated single spores were transferred to new PDA plates. The plates were cultured at 25 °C for 4 days to check for the presence of mycovirus using dsRNA extraction. All experiments were performed in five replicates. 

### 2.7. Effect of Mycovirus on Fungal Colony Morphology, Growth, and Pathogenicity

The morphology of the virus-free and virus-infected strains were observed through the radial growth by the measurement and photography of colonies after the cultures had grown for 5 days at 25 °C on PDA and on Vogel’s agar [32]. All experiments were performed independently in triplicate. 

For the assessment of conidial production, the virus-free and virus-infected strains were incubated for 10 days on PDA. Conidia were harvested as described above, filtered through cheesecloth, and counted using a hemocytometer. The experiment was repeated independently three times.

The diameter of lesions caused by fungal development has been used previously to determine virulence in mango (cv. Nam Dok Mai) and apple (cv. Scilate) [33]. Commercially available mango and apple fruits and twigs and young mango leaves collected from 2-year-old mango trees in an orchard in Ratchaburi, Thailand served as the materials for the pathogenicity studies. Briefly, fruits, leaves, and twigs were washed with tap water, surface sterilized with 75% ethanol, and then rinsed with sterile distilled water several times. The surfaces of fruit and twig samples were punctured with small holes (5 mm diameter) while needles were used to make a small hole on the leaf surfaces. Agar plugs from the growing margin of a 4-day-od colony were directly placed on the fresh wounds in each plant sample and then wrapped in Parafilm. All treated plant parts were placed into a plastic chamber lined with wet tissue paper, while the ends of each twig were covered with wet tissue paper to avoid desiccation. All samples were kept at 25 °C for 7 days. Following 2 days of inoculation, the parafilm was removed from the surface. Lesion sizes were measured 7 days post-inoculation, as indicators of disease development. Each sample was inoculated on five separate occasions.

### 2.8. Statistical Analyses 

For the growth rate, virulence and sporulation mean values for each biological replicate are presented with error bars representing standard deviation (SD). One-way analysis of variance (ANOVA) was performed to assess statistical significance and overall differences using IBM SPSS Statistics with *post hoc t*-tests. Outliers, when present, did not affect the outcome of the statistical analysis. *p*-values < 0.05 were considered to indicate statistical significance.

## 3. Results

### 3.1. Identification and Biological Characteristic of Virus-Infected Isolate Ssa-44.1

**Figure 1:**
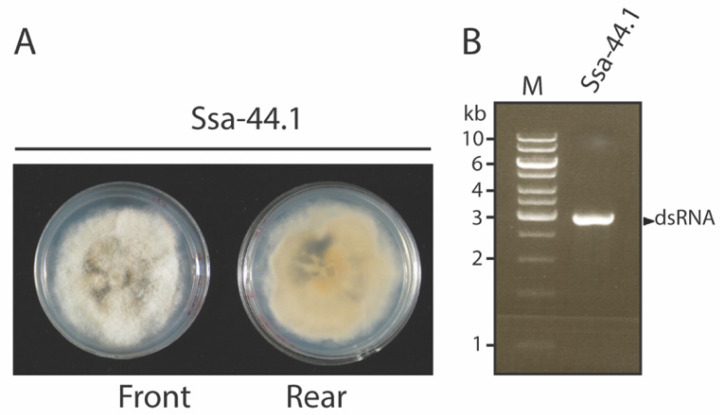
Colony morphology of anthracnose causing disease, *C. gloeosporioides*, and mycovirus dsRNA profiles. (**A**) Morphology of *C. gloeosporioides* strain Ssa-44.1 following 5 days incubation at 25 °C on PDA at 5 days after incubation. (**B**) Presence of mycovirus confirmed by gel electrophoresis on 1% agarose gel. Lane M, marker (GeneRuler 1 kb DNA Ladder).

Mango parts showing fungal infection were collected and identified from Phitsanulok and Saraburi, Thailand. Strain Ssa-44.1 was isolated from diseased mango parts showing anthracnose symptoms in Saraburi province. Based on cultural morphology, strain Ssa-44.1 exhibited a white to greyish cottony colony after five days of inoculation on PDA. Orange-colored conidiomata superficially developed primarily in the colony’s center. The fungal hyphae did not grow in an even fashion and the colony margin was irregular (Figure 1A). Conidia measured (n = 20) 12.8 to 16 × 4.4 to 6 μm (length/width) and were unicellular, aseptate, cylindrical to oblong with obtuse ends and occasionally slightly curved (Supplementary Figure S1A). Molecular identification using PCR detection with ITS primers was conducted to confirm the provenance of the host; the results showed that strain Ssa-44.1 was 99% identical to *Colletotrichum gloeosporioides* (Supplementary Figure S1B). To determine whether strain Ssa-44.1 contains a mycovirus, dsRNA isolation was conducted. Figure 1B shows a single dsRNA fragment with a size of ca. 3 kbp recovered from strain Ssa-44.1. The dsRNA nature of the nucleic acid was confirmed by its resistance to digestion by DNase I and S1 nuclease (Supplementary Figure S2). 

### 3.2. Genome Organization and Phylogenetic Analysis of Mycovirus Ssa-44.1

**Figure 2:**
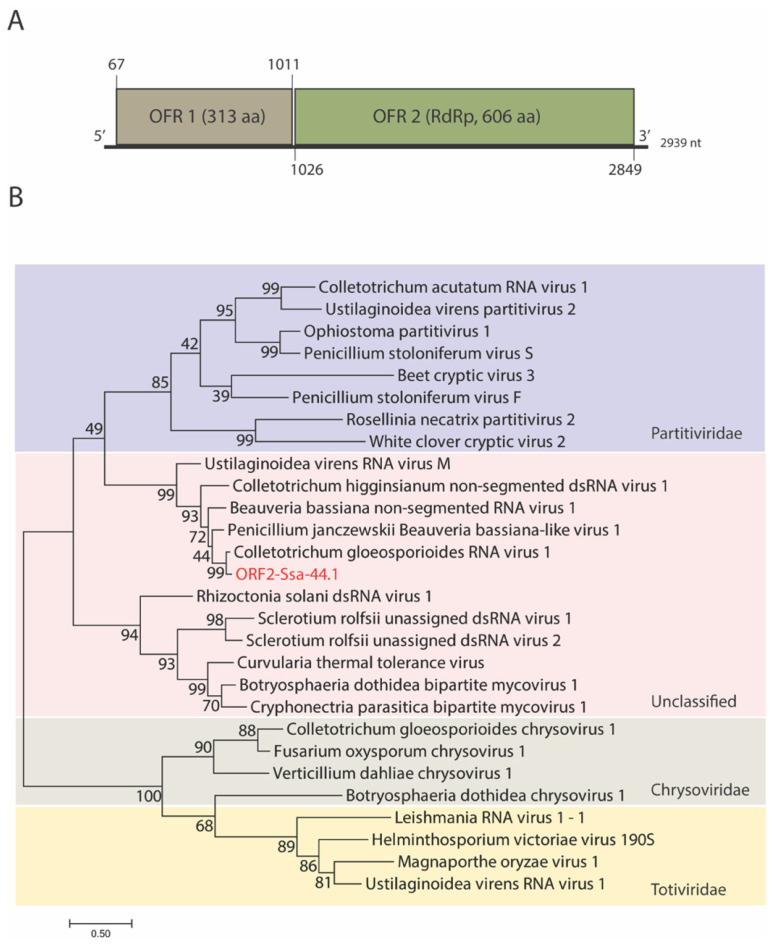
Schematic representation of mycovirus genome strain Ssa-44.1 and phylogenetic analysis, (**A**) The genetic organization of mycovirus Ssa-44.1 is depicted in this illustration. The genome of the mycovirus Ssa-44.1 is 2939 bp long and contains two ORFs (ORF-1 and ORF-2). The brown and green rectangles represent open reading frames (ORFs) encoding a hypothetical protein and the RdRp, respectively. (**B**) The maximum likelihood method was used to create a phylogenetic tree based on the alignment of complete RdRp aa sequence together with previously reported members of the genera *Partitivirus*, *Chrysovirus*, *Totivirus,* and Unclassified dsRNA viruses. The percentage of trees in which the associated taxa clustered together is shown next to the branches. In the phylogenetic tree, CgRV1-Ssa-44.1 ORF 2 encodes the RdRp and is represented by a red letter; genetic distance substitutions are displayed by scale bars.

To obtain the complete genome sequence of the suspected dsRNA mycovirus, next-generation sequencing and RACE-PCR were performed. The dsRNA of strain Ssa-44.1 is 2939 bp long, with a G + C content of 54.06%. Two ORFs were found on the coding strand of Ssa-44.1 dsRNA. ORF 1 (nt 67 to 1011) encodes a hypothetical protein containing 313 amino acids (aa), while ORF 2 (nt 1026 to 2849) encodes a 606-aa protein. A BLASTn search showed that the full-length dsRNA sequence of Ssa-44.1 was most closely related to Colletotrichum gloeosporioides RNA virus 1 (CgRV1) genome with the identity of 82.14% and was nominated Colletotrichum gloeosporioides RNA virus 1 strain Ssa-44.1 (CgRV1-Ssa-44.1). At the 5-and 3′-termini of CgRV1-Ssa-44.1 respectively, 66 and 90 nt untranslated regions (UTRs) were found (Figure 2A). The potential secondary structure of the 5′-and 3′-termini was predicted using Mfold RNA structure software. The 5′ UTR could be folded into a stem-loop structure with a ∆G of −6.80 kcal/mol and the 3′UTR with 90 nt was predicted to form a stem-loop with a ∆G of −45.30 kcal/mol (Supplementary Figure S3).

CgRV1-Ssa-44.1 ORF 1 encoded a hypothetical protein and exhibited 88.22% (E-value: 5 × 10^−168^ query cover: 100%) aa identity to a hypothetical protein of CgRV1 (QED88100) [28], followed by other unclassified dsRNA viruses including the capsid protein encoded by Beauvaria bassiana non-segmented RNA virus 1 [34] (BbNRV1; AZT88649.1; E-value: 2 × 10^−97^; query cover: 100%; identity 56.37%); hypothetical protein of Penicillium janczewskii Beauveria bassiana-like virus 1 (PjBlV1) [35] (PjBlV1; ALO50135; E-value: 3 × 10^−97^; query cover: 100%; identity 56.05%) and hypothetical protein of Colletotrichum higginsianum non-segmented RNA virus 1 [36] (ChNRV1; YP. 009177217.1; E-value: 5 × 10^−84^; query cover: 81%; identity 58.37%). 

The protein encoded by CgRV1-Ssa-44.1 ORF 2 showed 90.19% (E-value: 0; query cover: 97%) aa identity to the CgRV1 RNA-dependent RNA polymerase (RdRp) (QED88100) [28], followed by other unclassified dsRNA viruses including Penicillium janczewskii Beauveria bassiana-like virus 1 [35] (PjBlV1; ALO50135; E-value: 0; query cover: 96%; identity 70.55%), Beauvaria bassiana non-segmented RNA virus 1 [29] (BbNRV1; AZT88649.1; E-value: 0; query cover: 96%; identity 67.58%); Colletotrichum higginsianum non-segmented RNA virus 1 [36] (ChNRV1; YP. 009177217.1; E-value: 0; query cover: 93%; identity 57.47%) and Ustilaginoidea virens RNA virus M [37] (UvVM; YP_009094186.1; E-value: 2 × 10^−155^; query cover: 78%; identity 49.90%). 

Sequence analysis and Blast searches indicated that this dsRNA was the genome of an unclassified dsRNA mycovirus named Colletotrichum gloeosporioides RNA virus 1 strain Ssa-44.1 (CgRV1-Ssa-44.1). The sequence of the dsRNA was deposited in the GenBank under the accession number ON887156 and the complete sequence was provided in Supplementary Figure S4.

Phylogenetic analysis was performed using the aa sequence of the hypothetical protein and RdRp encoded by CgRV1-Ssa-44.1 ORF 1 and ORF 2, respectively, with other selected mycovirus aa sequences. The aa sequences were multiple-aligned and utilized MEGA 7’s Maximum Likelihood method was utilized to generate phylogenetic trees. CgRV1-Ssa-44.1 was placed in the same clade as several unclassified dsRNA viruses and has the closest relationship with CgRV1 the previously identified unclassified dsRNA virus (Figure 2B and Figure S5).

### 3.3. Biological Properties of CgRV1-Ssa-44.1 on C. gloeosporioides 

**Figure 3:**
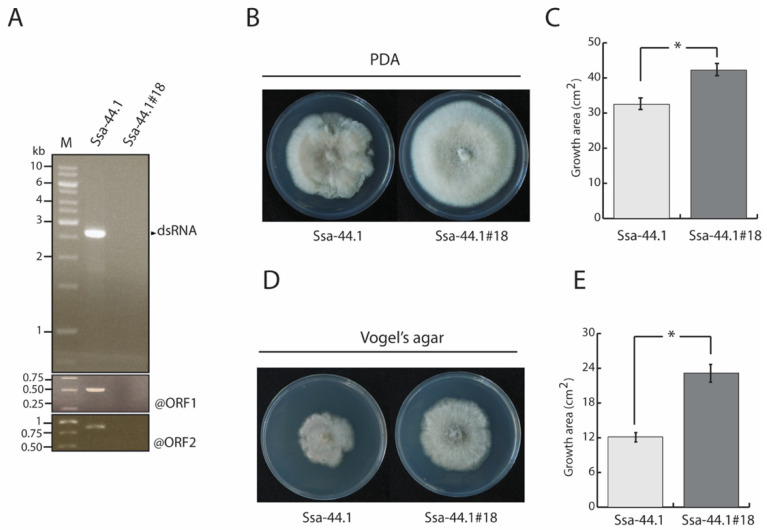
CgRV2’s dsRNA elimination and biological comparison between virus-infected and isogenic virus-free strains. (**A**) Confirmation of the presence of double-stranded RNA between virus-infected CgRV2 and isogenic virus-free strain Ssa-44.1#18 using 1% gel electrophoresis and RT-PCR methods with primers generated from ORF 1 and 2 of CgRV2 sequence. Lane M, marker (GeneRuler 1 kb DNA Ladder). (**B**–**E**) Colony morphology (**B**,**D**) and growth area (**C**,**E**) of fungi when grown at 25 °C on PDA (**B**,**C**) and Vogel’s agar (**D**,**E**) for 5 days. The bars indicate standard deviation from five replicates and asterisks indicate that there was significant difference at *p* < 0.05 using one-way ANOVA analysis (**C**,**E**).

Single spore isolation was used to eliminate mycoviruses from the strain Ssa-44.1. Single spores were grown on PDA for five days at 25 °C. dsRNA isolation was conducted for each fungal spore-derived colony and gel electrophoresis was performed to verify CgRV1-Ssa-44.1 elimination which was confirmed by RT-PCR amplification using primers generated from the CgRV1-Ssa-44.1 ORF 1 and 2 sequences. Figure 3A shows the absence of dsRNA in *C. gloeosporioides* isolate Ssa-44.1#18. The isogenic virus-free fungal isolate was nominated as *C. gloeosporioides* Ssa-44.1#18. These findings confirmed that the cured strain was mycovirus-free and was available for direct comparison with the virus-infected line. 

The colony morphologies of the virus-infected and virus-free *C. gloeosporioides* were compared following five days of growth on PDA agar at 25 °C; the latter appeared healthier compared to the former (Figure 3B). The colony hyphae of the virus-free isolate were denser and grew significantly faster compared to the virus-infected strain. The virus-free isolate growth area was significantly larger than its virus-infected counterpart (Figure 3C). Similar results were obtained when the isogenic lines were grown on Vogel’s agar (Figure 3D). Growth areas of 12.15 and 23.19 cm^2^ were observed on virus-infected and virus-free, respectively (Figure 3E). On nutrient-limited media (Vogel’s), the morphological differences between the two strains were more apparent than on PDA (Figure 3B,D).

To evaluate the effect of virus infection on fungal sporulation in *C. gloeosporioides*, spore counting was performed in triplicate. The results in Table 1 show that the virus-infected line produced significantly fewer spores compared to the virus-free line at 2.27 × 10^7 (a)^ and 4.86 × 10^8 (b)^ spores/mL, respectively (*p* < 0.05). These results suggest that CgRV1-Ssa-44.1 elicits a hypovirulent effect on the growth and sporulation of *C. gloeosporioides.*

### 3.4. Transmission of CgRV1-Ssa-44.1 in C. gloeosporioides

**Figure 4:**
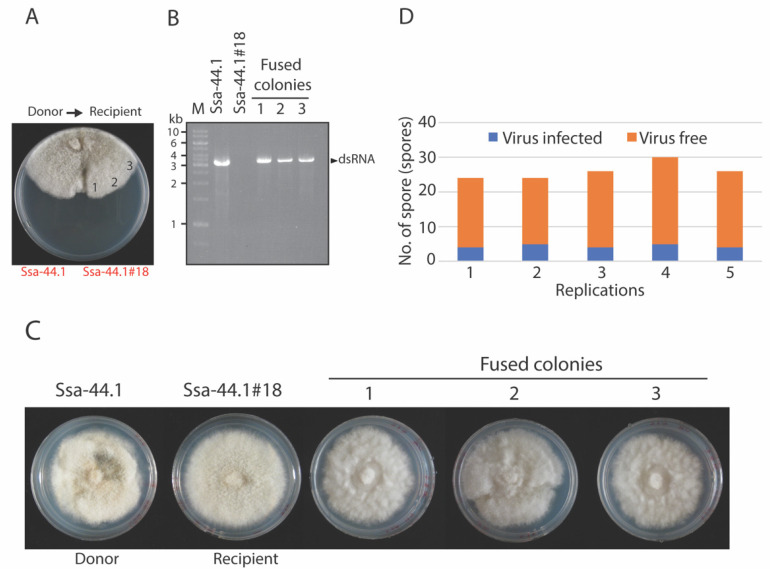
Transmission of CgRV1-Ssa-44.1 to *C. gloeosporioides.* (**A**) Horizontal transmission via co-culturing of virus-infected strain Ssa-44.1 as a donor and virus-free strain Ssa-44.1#18 as a recipient on PDA for 6 days at 25 °C. The transmission of the virus from donor to recipient fungus is indicated by an arrow. Number (1, 2, and 3) indicates the position where the mycelia of fungal recipients were taken for subsequent culture. (**B**) Double-stranded RNA fractionation on 1% agarose gel. Lane M, marker (GeneRuler 1 kb DNA Ladder). Each lane represents an independent fused colony. (**C**) Colony morphology of fungi when grown at 25 °C on PDA for 5 days. (**D**) Vertical transmission of CgRV1-Ssa-44.1 via spore. Each single spore isolate was checked for the presence of dsRNA; the figure shows the presence or absence of dsRNA in each replicate.

The cgRV1-Ssa-44.1 transmission was investigated by hyphal anastomosis with isogenic virus-free isolate SSA-44.1#18 (Figure 4A). Anastomosis is a mechanism where hyphae fuse to one another to connect and share the cytoplasm and nuclei [32]. The fusion of isolates was checked for the presence of dsRNA fractionated by electrophoresis and showed that CgRV1-Ssa-44.1 can be successfully transmitted through hyphal anastomosis (Figure 4B). All three of the fused colonies examined contained CgRV1-Ssa-44.1 dsRNA. The morphology of fused colonies was found to be similar to the virus-infected donor. They showed a smaller colony size compared to the virus-free strain (Figure 4C). Single spore isolation was used to examine CgRV1-Ssa-44.1 transfer into *C. gloeosporioides* and check frequency. Single spores were collected and checked for the presence of CgRv1-Ssa-44.1 dsRNA. The results revealed that the vertical transmission rate of CgRV1-Ssa-44.1 in *C. gloeosporioides* was 17.60 + 2.08% (22 out of 130 spores) (Figure 4D). These observations suggest the efficient horizontal and vertical spread of CgRV1-Ssa-44.1. 

### 3.5. Effect of CgRV1-Ssa-44.1 on Virulence of C. gloeosporioides

**Figure 5:**
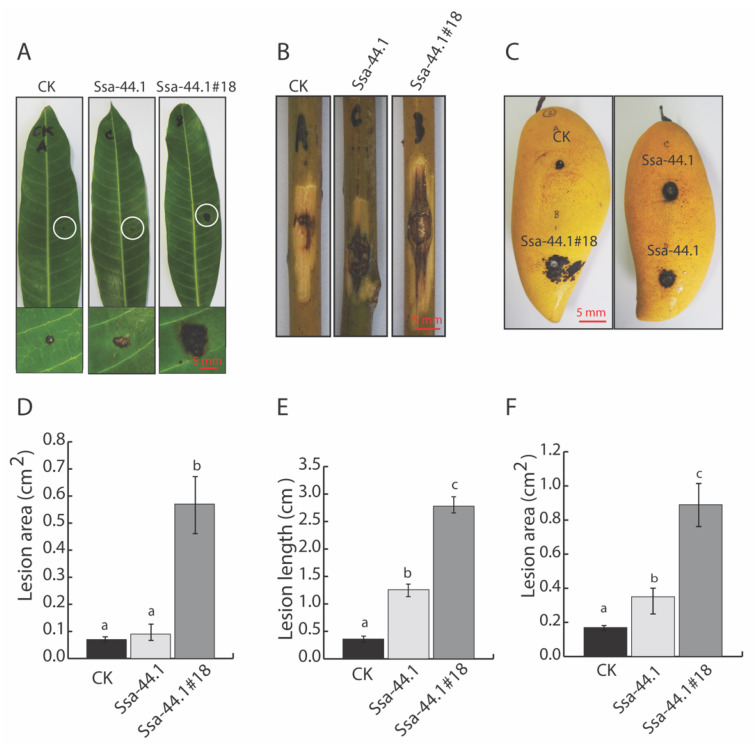
Virulence assay of virus-infected and virus-free *C. gloeosporioides* on mango. (**A**) The images illustrate representative lesions elicited by the fungal strains 7 days after mango leaf inoculation. A white circle was used to represent the symptom zones, which were enlarged at the bottom of each leaf image. (**B**) The images illustrate representative lesions elicited by the fungal strains 7 days after mango twig inoculation. (**C**) The images illustrate representative lesions elicited by the fungal strains 10 days after mango fruit inoculation. (**D**) Graphical representation of lesion areas on mango leaves measured as described in (**A**). (**E**) Graphic representation of lesion lengths on mango twigs measured as described in (**B**). (**F**) Graphic representation of lesion areas on mango fruits measured as described in (**C**). The data from 5 replicates are means ± SD with different letters that are significantly different (*p* < 0.05) among treatments using one-way ANOVA analysis. The control variable (CK) was inoculated with PDA (agar plugs) only.

The influence of CgRV1-Ssa-44.1 on the pathogenicity of *C. gloeosporiodes* was investigated. Both the virus-infected strain Ssa-44.1 and the virus-free strain Ssa-44.1#18 were inoculated on different parts of the mango and the diameters of the lesions that developed were measured. The diameters of anthracnose lesions that develop following inoculation with the virus-infected strain Ssa-44.1 on leaves, twigs, and fruits of mango were significantly smaller than the lesions elicited by the isogenic virus-free strain Ssa-44.1#18 (Figure 5). When both fungi were inoculated on mango leaves for 7 days, the isogenic virus-free strain Ssa-44.1#18 produced significantly larger black spot lesions ca. 0.57 cm^2^ compared to the virus-infected strain Ssa-44.1 which produced lesion areas of 0.09 cm^2^ (Figure 5A,D). Furthermore, the isogenic virus-free strain Ssa-44.1#18 also produced significantly larger lesions on mango twigs with a length of ca. 2.78 cm compared to virus-infected strain Ssa-44.1 which produced lesion lengths of ca. 1.26 cm (Figure 5B,E). Similar results were found following the inoculation of mango fruits and apples. For mango, the virus-free strain Ssa-44.1#18 elicited lesion areas of 0.89 cm^2^, while the virus-infected strain Ssa-44.1 produced smaller lesion areas of ca. 0.35 cm^2^ (Figure 5C,F).

With apple fruits, similar results to those found with mango were observed, where the virus-free strain Ssa-44.1#18 elicited a significantly larger lesion of 2.53 cm^2^ compared to the virus-infected strain Ssa-44.1, which produced a lesion area of 0.41 cm^2^ (Supplementary Figure S6). Our findings suggest that CgRV1-Ssa-44.1 confers hypovirulence in *C. gloeosporioides* hosts and can reduce pathogenicity on mango parts and apple fruit.

## 4. Discussion

Anthracnose disease, caused by *C. gloeosporioides*, is a major problem in agriculture around the world. It is currently troubling the agriculture sector, particularly mango orchards, by lowering its economic value. The current treatment for mango anthracnose relies heavily on the use of fungicides. However, the excessive use of agrochemicals has its downsides including contamination of the environment [38]. Alternatively, the use of mycoviruses as biocontrol agents may serve as a viable way to control economically important fungal diseases like chestnut blight disease and now in this study, mango anthracnose disease. *C. gloeosporioides* has been found to be infected by a few mycoviruses from different genera [25,26,27,28]. The incidence of *C. gloeosporioides* dsRNA mycovirus was expanded with the discovery of *C. gloeosporioides* strain Ssa-44.1, which was isolated from different mango parts collected from Saraburi province, Thailand (Figure 1). *C. gloeosporioides* strain Ssa-44.1 harbored a dsRNA element of 2939 bp, with 2 ORFs encoding for a hypothetical protein that is possibly a virus capsid protein and an RdRp. Through sequence alignment and phylogenetic analysis based on the RdRp mycovirus from strain Ssa-44.1, it was found to have high sequence similarity with Colletotrichum gloeosporioides RNA virus 1 (CgRV1) [28] with 82.14% nt and 90.19% aa sequence identity. Strain Ssa-44.1 belongs to a group of unclassified mycoviruses including CgRV1, BbNRV1, PjBlV1, and UnVM [28,34,35,36,37] which are distantly related to mycoviruses from the *Partitiviridae*, *Chrysoviridae* and *Totiviridae* families and we propose the name Colletotrichum gloeosporioides RNA virus 1 strain Ssa-44.1 (CgRV1-Ssa-44.1) (Figure 2). However, there are no data concerning the biological or virulence effects of mycovirus infection on *C. gloeosporioides*. Therefore, obtaining a virus-free strain is an important step in understanding the effects of mycoviral infection on the fungal host. Several techniques have been used to isolate virus-free strains including single spore isolation, hyphal tipping, protoplast isolation, and chemical treatment [39]. Using a single spore isolation approach, we were able to eradicate the mycovirus from strain Ssa-44.1. dsRNA extraction, agarose gel electrophoresis, and RT-PCR amplification were used to confirm that the dsRNA had been removed from cured fungal hyphae (Figure 3A).

Finding Mycoviruses is becoming more common in a variety of fungal families, including fungi that cause both animal and plant infections [21,40]. While many have little or no effect on their host fungi, others elicit phenotypic changes in the host, including hypovirulence and host debilitation, including decreased growth, abnormal coloring, altered reproduction, and diminished pathogenicity [21,41,42]. Hypovirulence, which has been researched extensively in the fungus *C. parasitica*, is an intriguing phenomenon for the biological control of fungal infections [17]. The effect of mycovirus infection on *C. gloeosporioides* was investigated for the first time in this study and revealed the presence of CgRV1-Ssa-44.1 in *C. gloeosporioides* which differed from its virus-free Ssa-44.1#18 isogenic line in that it changed colony morphology by significantly decreasing aerial growth on PDA and Vogel’s agar (Figure 3B–D). Moreover, the CgRV1-Ssa-44.1 virus suppressed *C. gloeosporioides* conidiation numbers (Table 1). Importantly, CgRV1-Ssa-44.1 dramatically reduced *C. gloeosporioides* pathogenicity in mango leaves, twigs and fruit (Figure 5) as well as in apple fruit (Supplementary Figure S4). For numerous fungal species, a link between the presence of dsRNA and hypovirulence has been established. Many fungal species, including several important plant pathogens, have been shown to have hypovirulence effects, including Botryosphaeria dothidea RNA virus 1 (BdRV1) in *Botryosphaeria dothidea* was found to cause hypovirulence in pear and apple fruits and shoots [43]. Sclerotinia sclerotiorum megabirnavirus 1 (SsMBV1) SsMBV1 has slightly impacted the biological properties of *S. sclerotiorum* [44]. Fusarium oxysporum ourmia-like virus 1 (FoOuLV1) was found to be associated with hypovirulence in *Fusarium oxysporum* f. sp. *Momordicae* [45]. Rosellinia necatrix megabirna virus 1 (RnMBV1) was found to be responsible for reducing virulence and mycelial proliferation in numerous *R. necatrix* host strains [18].

The transmission of mycoviruses is one of the key factors influencing their ecological fitness. Unlike animal and plant viruses, mycoviruses are transmitted in two ways: vertically and horizontally. The cgRV1-Ssa-44.1 virus is readily transmitted horizontally to isogenic virus-free strain SSA-44.1#18 via hyphal anastomosis (Figure 4A,B). Anastomosis occurs when hyphae from distinct fungal individuals fuse together, allowing genetic and cytoplasmic material to be exchanged [24]. Mycoviruses that thrive in the cytoplasm are similarly exchanged in the process. A heterokaryon is formed when filamentous ascomycetes fungi fuse to form a hyphal fusion, in which genetically distinct nuclei coexist in a shared cytoplasm [46]. The formation of heterokaryons potentially allows the transfer of genetic material without meiosis or a parasexual cycle [46]. The generation of heterokaryons is tightly controlled by *het* loci. If the individuals differ in specificity at one or more *het* loci, heterokaryotic fusion cells result in death by lytic lysis [47]. Aside from horizontal transmission, the efficacy of vertical transmission via asexual spores is another factor that assists mycovirus spread. In our study, CgRV1-Ssa-44.1 was less efficiently transmitted through conidia at 17.60% (Figure 4C). Vertical transmission efficiency has been found to differ between fungal isolates in several fungal species-mycovirus combinations [48]. For example, CHV1-EP713 is nearly 100% transferred through conidia in *C. parasitica*, but Mycoreovirus 1 (MYRV-1) is only about 10% transmitted through conidia [31,49]. Melanconiella theae mitovirus 1 (MtMV1) is 100% transmitted through all conidia of *Melanconiella theae* strain WJB-5 [43]. Some mycoviruses, such as Colletotrichum camelliae filamentous virus 1 (CcFV-1), were not found in the sub-isolates, implying that vertical transmission via conidia was not possible [50]. Pestalotiopsis theae chrysovirus-1 (PtCV1) showed 67% transmission through conidia [51].

In conclusion, this study characterized a new dsRNA mycovirus CgRV1-Ssa-44.1 in *C. gloeosporioides*. CgRV1-Ssa-44.1 can confer hypovirulence in its host *C. gloeosporioides* and could be successfully transmitted via hyphal fusion. However, the transmission via asexual spores was found to be less efficient. CgRV1-Ssa-44.1 has a close phylogenetic relationship with CgRV1. Therefore, CgRV1-Ssa-44.1 may be a candidate for use as a biological control agent against *C. gloeosporioides*. This study is by far, the first to report the biocontrol trait of a mycovirus from *C. gloeosporioides* isolated in Thailand.

## Figures and Tables

**Table 1:** Effect of CgRV2 on sporulation level of *C. gloeosporioides*.

Strains	Spore Counting (Spores/mL)			
	1	2	3	Mean ± SD *
Ssa-44.1	2.58 × 10^7^	1.85 × 10^7^	2.38 × 10^7^	2.27 ± 0.38 × 10^7 a^
Ssa-44.1#18	4.25 × 10^8^	5.39 × 10^8^	4.95 × 10^8^	4.86 ± 0.57 × 10^8 b^

* The results are shown as mean ± standard deviation of the three replicates (n = 3). Letters indicate significantly different values using one-way ANOVA analysis (*p* < 0.05).

## Data Availability

Not applicable.

## Supplementary Materials

The following supporting information can be downloaded at: https://www.mdpi.com/article/10.3390/v14091921/s1, Table S1: List of primers used in this study; Figure S1. Conidia morphology and Phylogenetic tree; Figure S2. Analysis of the CgRV1-Ssa-44.1 dsRNA segments; Figure S3. Predicted secondary structure of the 5′-and 3′-untranslated regions (UTRs) of CgRV2 with a complete genome sequence identified in *C. gloeosporioides*; Figure S4. Phylogenetic analysis of ORF 1 encoded hypothetical protein; Figure S5. Whole genome sequences of CgRV1-Ssa-44.1; Figure S6. Virulence assay of virus-infected and virus-free *C. gloeosporioides* on apple.
