# Supplementary material for: Hypovirulence of Colletotrichum gloesporioides Associated with dsRNA Mycovirus Isolated from a Mango Orchard in Thailand
Source: Viruses. 2022 Aug 30;14(9):1921. doi: 10.3390/v14091921 (PMC9504431; doi:10.3390/v14091921)
Supplement: Supplementary file 1 [file viruses-14-01921-s001.zip › viruses-1847547-supplementary.pdf]

**Table S1.** List of primers used in this study.

| Primer name | Sequences (5′-->3′) | Note |
|---|---|---|
| ITS1 | TCCGTAGGTGAACCTGCGG | ITS region of fungal strain |
| ITS4 | TCCTCCGCTTATTGATATGC | |
| 5RACE_545-P1 | TCTATCAAGGTTCGGAGC | 5′ RACE |
| 5RACE_398-P2 | GACTTCTGTGCGATTCCTAAC | |
| 3RACE_2213-P1 | CTCAATGGCTTATCTGGTGTT | 3′ RACE |
| 3RACE_2335-P2 | AGACCTTGAGGGAAATGG | |
| ORF1-357-F | CAAGATTACGGTTGACGATG | RT-PCR of ORF 1 |
| ORF1-866-R | AGTAGTTGGGTTCTCCGCA | |
| ORF2-1374-F | CGATTAGTCAACAGGATGGTG | RT-PCR of ORF 2 |
| ORF2-2179-R | CACTTCTCTGGCTTGAGCA | |

A

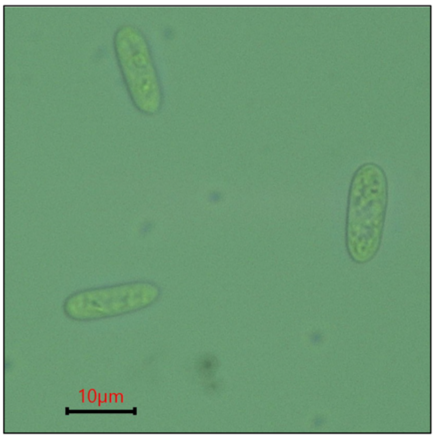


B

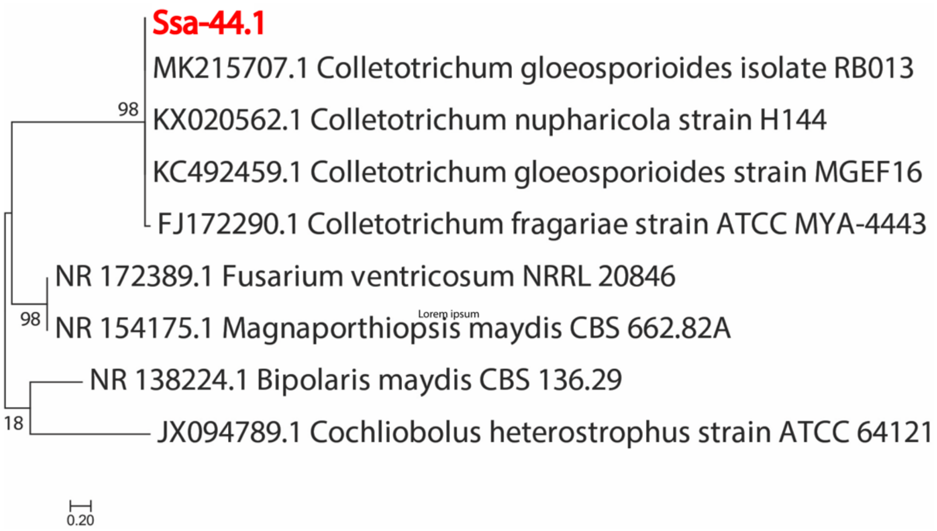


**Fig. S1.** Conidia morphology and Phylogenetic tree. (A) microscopic views of individual spores of *C. gloeosporioides* strain Ssa-44.1. Spore was observed under light microscope (40X). (B) Phylogenetic tree of isolated phytopathogenic fungal strain Ssa-44.1 and other related species, based on ITS gene partial sequences. Branch length values were shown, the tree was reconstructed using the Maximum Likelihood method and tested by bootstrapping (1,000 replicates). In the phylogenetic tree, strain Ssa-44.1 is represented by a red letter and substitutions per position are displayed by scale bars.

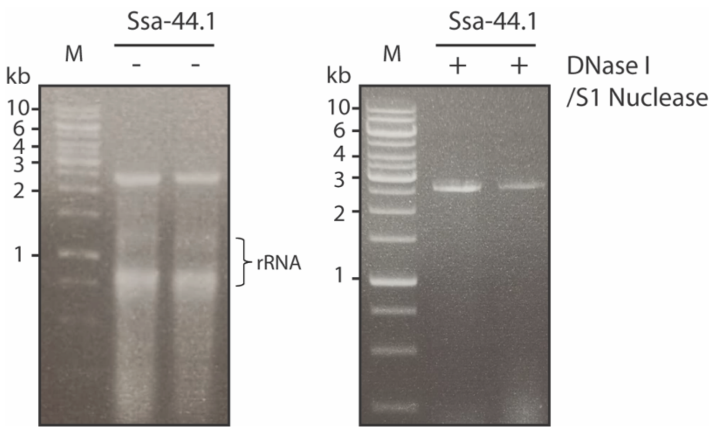


**Fig. S2.** Analysis of the CgRV1-Ssa-44.1 dsRNA segments. Electrophoretic profiles on a 1% aga-rose gel of dsRNA preparations from total RNA of strain Ssa-44.1 before (−) and after (+) digestion with DNase I and S1 nuclease.

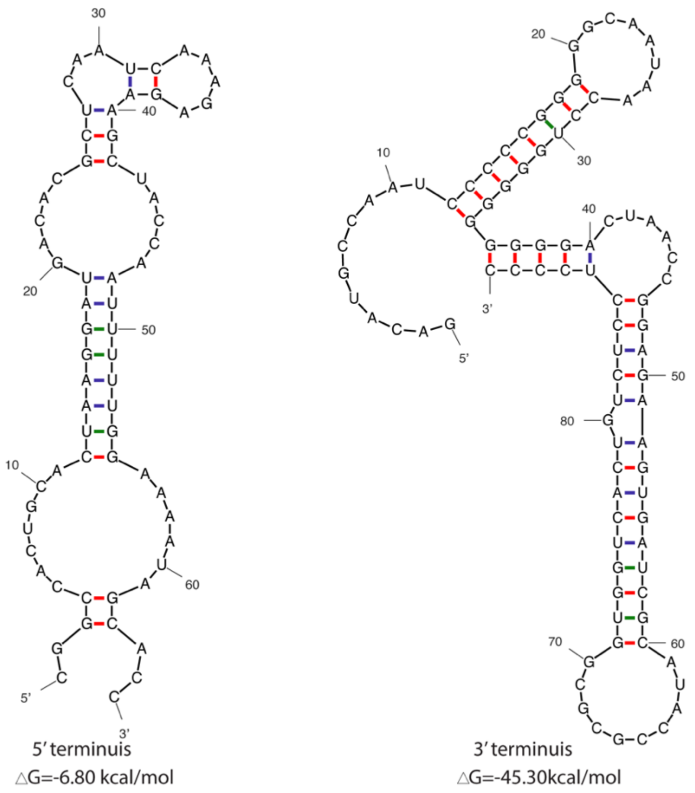


**Fig. S3.** Predicted secondary structure of the 5′-and 3′-untranslated regions (UTRs) of CgRV2 with a complete genome sequence identified in *C. gloeosporioides*. (A) Predicted secondary structure of the 5′-UTRs. (B) Predicted secondary structure of the 3′-UTRs. The RNAs were folded and the free energy (ΔG) was estimated using the Mfold web server (http://mfold.rna.albany.edu/?q=mfold/download-mfold) (accessed on 17 May 2022).

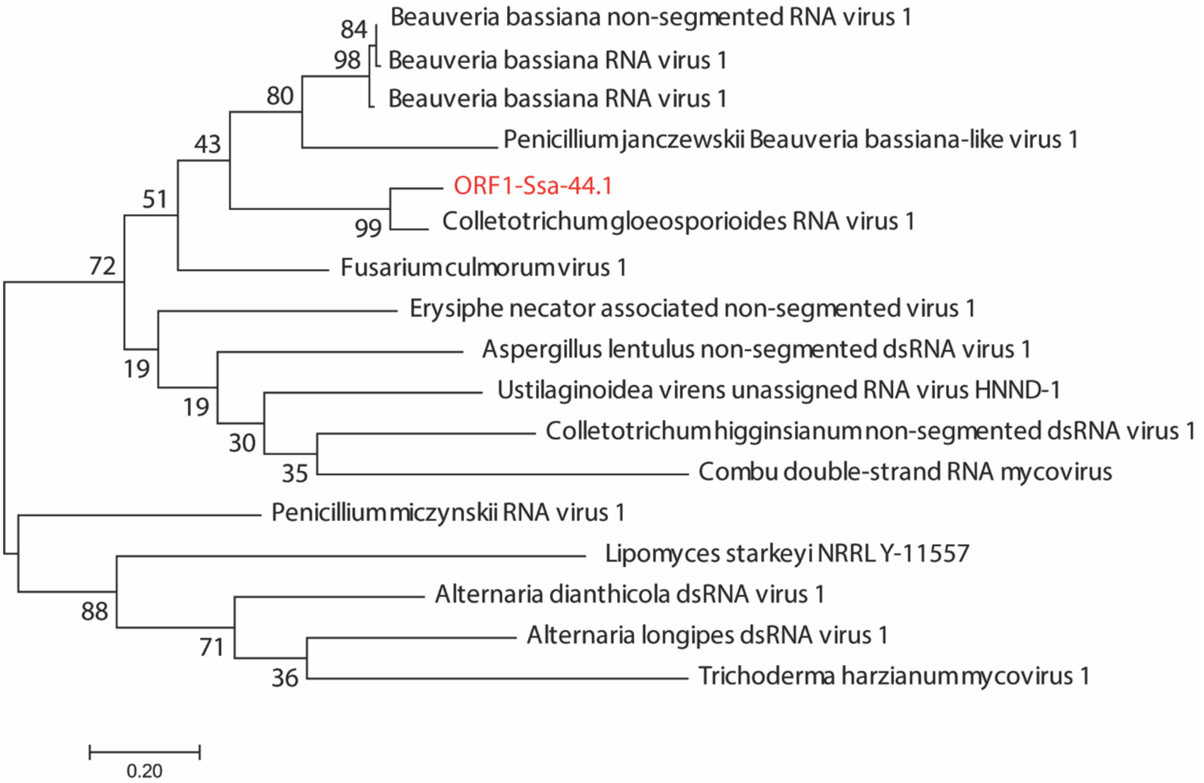


**Fig. S4.** Phylogenetic analysis of ORF 1 encoded hypothetical protein. The Maximum Likelihood method was used to create a phylogenetic tree based on the alignment of hypothetical protein complete aa sequence along with the previously reported members of other genera. The percentage of trees in which the associated taxa clustered together is shown next to the branches. In the phylogenetic tree, ORF 1 of. strain Ssa-44.1 encodes a hypothetical capsid protein is represented by a red letter and genetic distance substitutions are displayed by scale bars.

```
CGGCCACTGC ACTAAGGATG ACACGCTCAA TCAAAGAGAA GCTACCAATT TTTGGAAAAT    60
AGCACCATGG CCCCCAAACG AAATGTTGGG GATTTGGCGG AAGCTAGCTC TGGACGAGAT   120
CAGCCCCAGA GCGCCCCCGG TACCGCCCAA AATACCGAGG GAAGCCAGGC TGGCACTAGC   180
GCAGAACGCG CCCCGTCGCG TAGTCGACGT CCCGCCTTCA GGGCCCCAGA CCCTGAAGAG   240
TCACTCACCC GTAACGCGTA CCTAACTGGA ACGTCTTCGG GTCACGCAGC AGCAGTTCTC   300
TATGTCATAA GAGAATTCCT GGCGCAGCAA GGGGTTACCG ACCTGCCAGC AGACCCATCC   360
TCGCTCTTCC CGCAGGGATG GAAAGTTGGC AACACCCCTG TTCCAGTCCG CACTACTGGT   420
AACGACTTTC CTTGGCTGAG GGATGCCCTA CCTCATCTAC ATGGGTATGA CGCTTCAGGC   480
CAAGAGGTGC ACTGGATTGG TGAGGGCAGA GACATGTCCT CTGTTGTCCG TCTCACGGCG   540
AACTGGGATT CCCCCGTGTC CATTGTCTCC TGCATCTTGT CAGACACCAA GAATGTCAAG   600
ATTACGGTTG ACGATGTGTT AGGAATCGCA CAGAAGTCGC CTCAGGCTCT GGAGAAGCTT   660
AAGGCTTCCG GGAAGGAGTT TAAGGCCCAT CTTCAGCGAT TTCTCGACCC GGTGGCGTTG   720
AGACGGAGCC CCGAGTTTAA TAGAGTCTCT GCCGAGTACA AGGCTAAGCT CCGAACCTTG   780
ATTGATGCAC AGCAGAAGGC CATCATGGCC GCTCGCCAGG CGACAGCCGC CGTCAACAAG   840
TGCCTTGGAG AGAGGGATGA TGCCTTATCG TCCCTTGATC CTGGCTACGT CGCTAAGCGA   900
GCCACCGCAC GTAACGCACT AGCTGCTTAC GGCATTGATC TCGGTGAAGA TGCTGAGATG   960
GCAGATGCTG AGGAGACTGC GGCGCAGGCT CCTCAGGACT TGGATTTTTA GTTTCCGCGG  1020
CCTCGATGCT CGGCGGCGCG GAGTTGGGTG TAAGCGACAT TAATGAGGAT GTAATGGTCG  1080
ATTGGATTGC GGAGAACCCA ACTACTATGT CGGAGGCTAG CGGGAAGGGG ACTAAGGATT  1140
GGCGTTATTT TGTTTCGAAT AAGCACTGGC AGCGCCAGAT TCCAGGAGTT CAGGTTGTTG  1200
GATTTTCACC GGCCGTCAGG TATCGCTATG TTACACCTGA GACATCGGAT TCCTGGTACT  1260
TTTCGTCGTT TCTTGATGAG AGCGGGATTG AGCTGGGTGG CATTGAAGGC CCTGCCGCGG  1320
AGGTTATTAT GTCAGAGTTC GTTCCACCGT CCCAAGAGGA TCTTTATCAA CACATTGCCG  1380
GCTTTGGGGC CCCCGGTCCT GACGGGTCAC ACCTATCGAC TTACGATATC GCGCGAGTCC  1440
TTAAGGACTT ACATCGTGTC GAAAGTTGGG GTCCTCAGAT CGAGGGCCTC CTAGACGTGC  1500
AGCGTTTGCC AAAGATAAAG GTTCCTTCAC GTACCAGTCC TGGGATAAGG TGGAAGCGTC  1560
TCGGCTATAA GACCAAACGT AGCGCCCTGG TTCAGGCCGT GGTCGAATCA ACGCGATTAG  1620
TCAACAGGAT GGTGGAGGAG GGTCAGGAGT ATGACGTCCC CCCGTGTGGC GTCGCCGGGA  1680
GGGGGAAGAG GGTCGATATT AATCGTGACT TGACCGCGTC CGACCGCAAG GAGGGCAGAC  1740
TGATTGTCAT GCCTGATCTA GTGCGCCACT TAATCGGCTC GCTTGGATCA TCCCCTTATA  1800
TGGCGAGATT AAGGAACCTC GATCACTCTC TCGGTGGCGT CCTCTTGGGC ATGGGCCCAT  1860
TTCAAGAGCA GTATCAGAGT ATTGCTGAAT GGGCTTCCGG AGCGAACCAT TTCCTGTTCA  1920
TCGATTTCAA GAAGTTTGAT CAGCGCATTC CCCGCAGACT TCTACAAGTA GTCATGAAGT  1980
ATATTGCAGG AAGGTTCACA GCCGGCCCCG GAACGCGAGC GTATTGGAAG TCTGAGTTTC  2040
GCCACCTGGT CGAAACTGAG ATCGCGATGC CGGATGGATG CGTGTACAAG AAGAAGAGGG  2100
GCGTCGCCTC CGGTGACCCA TGGACTTCCC TAGCGGACTC ATATGCCAAT TATATAATCC  2160
TCAAGATGGC ATGTGATAAG CTGGGGCTCA AGGTTAAGAT TTGGACGTTT GGGGATGATA  2220
GCGTTGTTGC TGTATGGGAT TGGAAGTCCC AGTACGACTT GTTGGCTGAG GTTTCGAGGA  2280
TTGCATGGGA CATGTGGGGG ATGGTAACTA GTAAAGAAAA GAGCTACTCT AGCTCACATC  2340
TAGTGGACAT CGCAGAGAAT CCCGAACCAC GTAAGGGGGG TTCTTTTCTG TCGATGTATT  2400
TCCTACAAAC AGAAATGGGG GTTCGGCCGA CTAGGCCCCT CCAGGACATG TATGAGCTCA  2460
TGCTCAAGCC AGAGAAGTGC AGAAACTCGC TGGAGTGGGA GGTCGTACGA ACCTCAATGG  2520
CTTATCTGGT GTTCTACTAC AATAGCGACG CCAGATTCGT CCTTGAGGAG TATTGGACCT  2580
GGCTCCACCG CAAGTTCAAA ATTCCCGAGT TGACTGGCAC GGCAGAAGAC TTGGAGACCT  2640
TGAGGGAAAT GGACATTCCA TGGTCCTCCT TCAAGATGGA ATGGCTGAAT CGCCTACCTT  2700
TCAGTGGCGA GGTAGAGCTG ATGTATAAAT ATGGCCACAC AAAGTTCTTC GCTCCTGTTT  2760
TGTGGGCAAC GTGGTATCAT GGAGACGGGG ATGTGTTGGG CAACCACCTC GTTAATGATC  2820
CACGCCTGTA CGTAGACCCA CCAGATTAGG ACATGCCAAT CCCCCGGGGG CAATAACCTG  2880
GGGGGGGGAC TAACCGGAGA AGTGATCGCA TACCGCGCGG TGGTCACTGT CTCCTCCCC   2939
```

**Fig. S5.** Whole genome sequences of CgRV1-Ssa-44.1.

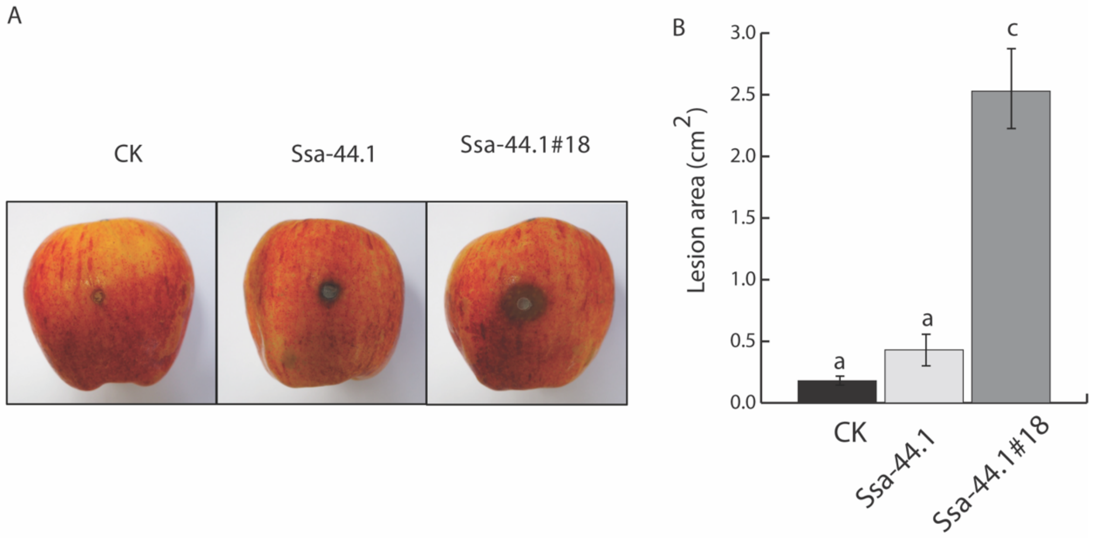


**Fig. S6.** Virulence assay of virus-infected and virus-free *C. gloeosporioides* on apple. (A) The images illustrate representative lesions elicited by the fungal strains 7 days after apple inoculation. (B) Graphical representation of lesion areas on apple measured in the experiment described in (A). The data from 5 replicates are means ± SD with different letters are significantly different ($p<0.05$) among treatments using one way ANOVA analysis. Control variable (CK) was inoculated with PDA (agar plugs) only.
